# Intestinal Morphology in Broiler Chickens Supplemented with Propolis and Bee Pollen

**DOI:** 10.3390/ani9060301

**Published:** 2019-05-31

**Authors:** Ivana Prakatur, Maja Miskulin, Mirela Pavic, Ksenija Marjanovic, Valerija Blazicevic, Ivan Miskulin, Matija Domacinovic

**Affiliations:** 1 Faculty of Agrobiotechnical Sciences Osijek, Josip Juraj Strossmayer University of Osijek, 31000 Osijek, Croatia; ivana.prakatur@fazos.hr (I.P.); matija.domacinovic@fazos.hr (M.D.); 2 Faculty of Medicine Osijek, Josip Juraj Strossmayer University of Osijek, 31000 Osijek, Croatia; maja.miskulin@mefos.hr (M.M.); ksenija.marjanovic@mefos.hr (K.M.); 3 Faculty of Veterinary Medicine, University of Zagreb, 10000 Zagreb, Croatia; mirela.pavic@vef.hr; 4 Faculty of Dental Medicine and Health Osijek, Josip Juraj Strossmayer University of Osijek, 31000 Osijek, Croatia; valerija.blazicevic@gmail.com

**Keywords:** intestinal morphology, duodenum, intestinal villi, intestinal absorption, broilers feeding, propolis, bee pollen

## Abstract

**Simple Summary:**

Consumers are becoming more aware of the nutritional value of foods, and they want to consume food that provides health benefits beyond the provision of essential nutrients. Chicken meat could fulfil the above requirements due to its high nutrient content and relatively low caloric value, and it serves as an interesting basis for functional foods. In this study, we evaluated the effects of propolis and bee pollen, as potential additives, on the intestinal morphology and absorptive surface areas of broiler chickens. The results of this study showed that supplementation of broilers with propolis and/or bee pollen has a profoundly beneficial effect on intestinal morphology and absorptive surface areas. Thus, these natural additives could be used as alternative additives in modern broiler production, while chicken meat can be even more beneficial for human health.

**Abstract:**

The aim of this study was to determine the influence of dietary supplementation with propolis and bee pollen on the intestinal morphology and absorptive surface areas of chickens. Two hundred day-old Ross 308 chickens (100 male and 100 female) were equally allocated into five groups. Throughout the whole study, the control group of chickens was fed with a basal diet, while the experimental groups of chickens were fed with the same diet supplemented with propolis and bee pollen: P1 = 0.25 g of propolis/kg + 20 g of bee pollen/kg; P2 = 0.5 g of propolis/kg; P3 = 1.0 g of propolis/kg; P4 = 20 g of bee pollen/kg. The duodenal villi of chickens from all experimental groups were significantly higher and wider (*p* < 0.001), while their duodenal villi crypts were significantly deeper (*p* < 0.001) in comparison with these parameters in chickens from the control group. The villus height to crypt depth ratio, as well as the absorptive surface areas of broiler chickens, were significantly increased (*p* < 0.001) in experimental groups of chickens in comparison with the control group. These findings suggest that dietary supplementation with propolis and bee pollen has a beneficial effect on broilers chickens’ intestinal morphophysiology.

## 1. Introduction

Propolis and bee pollen belong to a group of natural substances of animal and vegetable origin with intense antioxidant and antimicrobial properties [1]. The bioactive components of propolis and bee pollen include flavonoids, phenolic acids and their derivatives, which are also responsible for the bactericidal, antiviral, antifungal, analgesic, anti-inflammatory, antioxidant, immunostimulating and immunomodulating effects of these compounds in humans and animals [1,2,3].

A large number of previous studies have suggested an increase in the production performance of chickens fed with propolis and/or bee pollen [4,5,6,7,8,9]. These effects could be related to the effect of propolis extract on gastrointestinal microbiota, which increases levels of beneficial bacteria and decreases pathogenic types [10]. This modulation of microbiota could promote intestinal health, since the beneficial bacteria could provide improved feed digestibility and protection against pathogens via competitive exclusion through a variety of mechanisms [11,12].

With consideration of the above, and also the fact that the European Commission banned the use of antibiotics as growth agents in 2006 [7,13], the use of natural feeding additives such as propolis or bee pollen is very important in terms of improvement of performance, health status and immune systems in broiler chickens [1,14].

The small intestine is an important organ responsible for the digestion and absorption of nutrients from the diet. Any changes in its function affect the function of other organs and systems in the organism [15]. There are only a few studies that have previously evaluated the effect of propolis and bee pollen on the intestinal morphology of broiler chickens, and their results are contradictory [12,16,17,18,19]. However, some of them have proven that these natural additives improved intestinal morphophysiology [16,17,18,19].

The aim of this study was to determine the influence of dietary supplementation with propolis and bee pollen on the intestinal morphology and absorptive surface areas of broiler chickens. 

## 2. Materials and Methods 

### 2.1. Animals and Diets

A total of 200 (100 male and 100 female) day-old Ross 308 broiler chickens were evenly distributed by gender for use in the present study. The feeding trial of the broilers was carried out on a farm in Eastern Croatia under the supervision of the Division for Animal Production and Biotechnology, Faculty of Agrobiotechnical Sciences Osijek, Josip Juraj Strossmayer University of Osijek. The experimental protocol was approved by the Committee for Animal Welfare of the Faculty of Agrobiotechnical Sciences Osijek, Josip Juraj Strossmayer University of Osijek (Approval code: 602-04/18-01/01; 2158-94-02-18-01).

The experiment was a completely randomized design, and broilers were allocated into five dietary treatments with two replicate groups of 20 birds per pen (5 diets × 2 replicates). The groups of broilers were housed under the same conditions during the whole experimental period. Temperature, humidity, and lighting in the facility were maintained within the optimum limits according to the manufacturer’s recommendations for the Ross 308 hybrid [20]. Breeding was conducted on wooden sawdust (10 cm depth) and lasted for six weeks (42 days). During the study, feed and water were offered to broilers ad libitum. For ensuring effective monitoring of all the investigated indicators, all the broilers were marked with a leg ring on the seventh day of the feeding trial.

During days 1–21 of the study, broilers were fed a mixture of broiler starter. During days 22–42 of the study, broilers were fed a mixture of broiler finisher. The composition and calculated analyses of feed mixtures used in the feeding of the broilers are shown in Table 1. Throughout the study, the control group (K) of the broilers was fed a standard diet without additives, while the experimental groups of broilers (P1, P2, P3 and P4) were fed the same diet supplemented with propolis and/or bee pollen: the P1 group was offered a diet supplemented with 0.25 g of propolis and 20 g of bee pollen per kg of diet; the P2 group was offered a diet supplemented with 0.5 g of propolis per kg of diet; the P3 group was offered a diet supplemented with 1.0 g of propolis per kg of diet; the P4 group was offered a diet supplemented with 20 g of bee pollen per kg of diet. The doses of bee pollen and propolis were selected on the basis of known broiler chicken gastrointestinal tract physiology and through series of pilot studies on a small number of animals. The inclusion of propolis and bee pollen into the feed mixture was performed using a vertical mixer (Briketstroj Ltd., Valpovo, Croatia).

Samples of raw propolis and bee pollen used in this study were obtained from apiaries located in naturally preserved areas of continental Croatia (around the city of Osijek, Eastern Croatia). Propolis and bee pollen were crushed mixed, in powder form, with dry feed mixture using a vertical mixer. Bearing in mind that the biological activity of propolis and bee pollen depends on the components of polyphenolic fraction, mainly flavonoids, in the propolis and bee pollen samples used in this study, the amount of total flavonoids (expressed as equivalents of quercetin) was determined by a colorimetric method according to Chang et al. [21]. The results of this analysis are shown in Table 2. The analysis was performed at the Department of Health Ecology within the Croatian Institute of Public Health in Zagreb, Croatia accredited according to HRN EN ISO/IEC 17025:2000.

### 2.2. Sample Collection, Measurements and Analysis

At the end of the feeding trial (i.e., day 42), 10 birds from each group were randomly selected and slaughtered for a necropsy examination. Fifty duodenal samples (10 from each group) were collected from the birds directly after slaughter and fixed in 10% neutralized formalin. The duodenal samples were 2 cm long and dissected at the midpoint of the duodenum. The fixed tissue samples were transported to the Department of Pathology and Forensic Medicine, Faculty of Medicine Osijek, where they were further processed. The tissues were then dehydrated with increasing concentrations of ethyl alcohol (70%, 90%, 96% and 100%), cleared in xylene and embedded in paraffin. The paraffin blocks were then cut using microtome, into four 5-μm-thick discontinuous paraffin-embedded sections per broiler duodenal sample that were stained with hematoxylin and eosin (H&E) and examined under a light microscope (Olympus CX40), while representative fields were photographed and digital images were captured for morphometric analysis. A computer morphometric program, Quick Photo Micro 3.0, was used for morphometric measuring the duodenal villi height and base width of the villi. The same computer program was used for measuring the duodenal villi crypt depth. For the measurement of duodenal villi height, cross-sections of 10 villi were randomly selected. The criterion for villus selection was based on the presence of intact lamina propria. Villus height and width, as well as crypt depth, were measured at 40× the objective magnification. The villus height was measured as the distance from the apex of the villus to the junction of the villus and crypt [22]. The villus width was measured as the distance from the junction to the basement membrane of the epithelial cell at the bottom of the crypt at the bottom third of the length of the villus (base width of the duodenal villi) [23]. All the measurements taken from 10 villi per one sample were counted from four different preparations from each duodenal segment for each bird, and were expressed as the average duodenal villi height and average base width of the duodenal villi for each bird. Finally, 10 average heights of duodenal villi, as well as 10 average base widths of the duodenal villi from 10 birds were expressed as the average height of the villi for a group and the average base width of the villi for a group [22]. The duodenal villi crypt depth was measured from the base of the villus to the mucosa [23]. All the measurements from 10 crypts were counted from four different preparations from each duodenal segment for each bird. Averaged depth measurements of 10 crypts were expressed as the average duodenal villi crypt depth for each bird. Finally, 10 average depths of duodenal villi crypts of 10 birds were expressed as the average depth of duodenal villi crypts of the group [22]. The ratio of villus to crypt was estimated by dividing the villus height by the crypt depth [23]. The absorptive surface area of the duodenal villus was estimated by considering a villus as a cylindrical structure [23]. Villus absorptive surface area was calculated using the formula: Villus absorptive surface area = 2π × (average villus width/2) × villus height [23,24].

### 2.3. Statistical Analysis

The normality of the data distribution was tested by a Kolmogorov–Smirnov test; all data were processed by methods of descriptive statistics. The numerical variables were described as the median and interquartile ranges. A Kruskal–Wallis test was used for the comparison of numerical variables among the groups. The level of statistical significance was set at *p* < 0.05. Statistical analysis was performed using the statistical package Statistica for Windows 2010 (version 10.0, StatSoft Inc., Tulsa, OK, USA).

## 3. Results

Morphometric analysis of the duodenal villi of broiler chickens revealed differences between the control and experimental groups of chickens at the tissue structure level on the 42nd day of the feeding trial, as shown in Table 3. The duodenal villi of chickens from all the experimental groups were significantly higher (*p* < 0.001), while their base was significantly wider (*p* < 0.001) in comparison to those in chickens from the control group. There was a statistically significant difference in duodenal villi crypt depth between the groups of chicken (*p* < 0.001).

**Figure 1:**
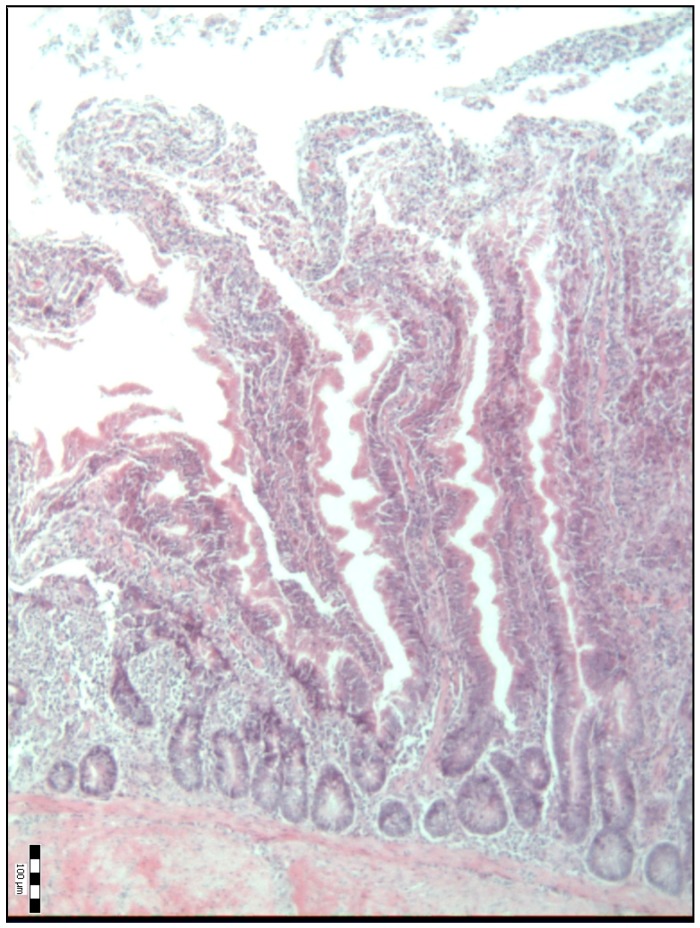
Histological representation of the duodenal villi of broiler chickens from the control group of chickens (K) (H&E; ×100).

**Figure 2:**
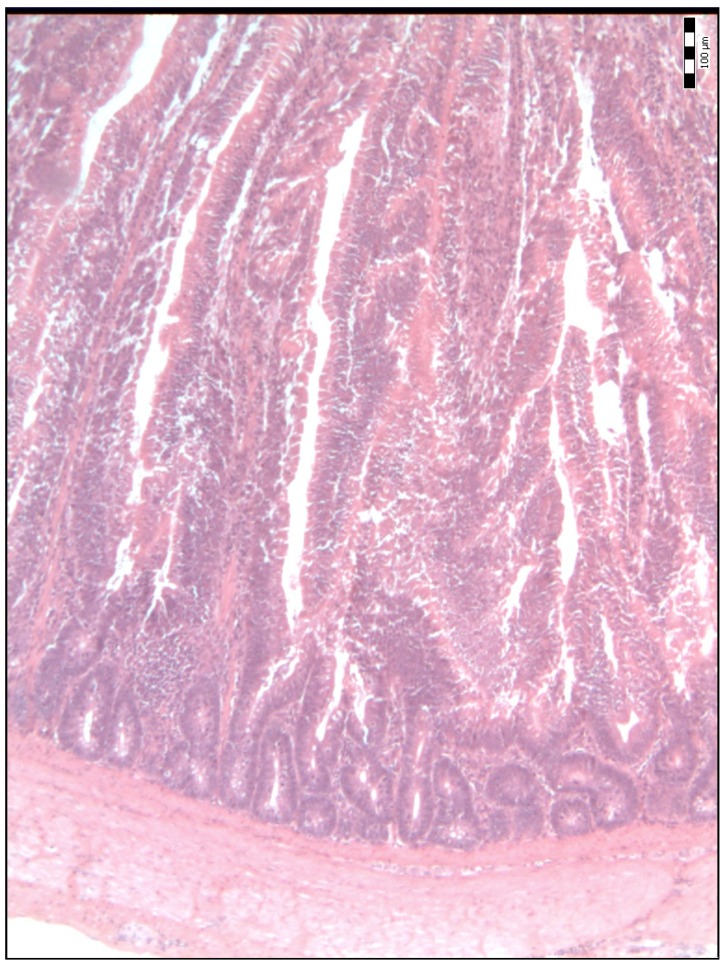
Histological representation of the duodenal villi of broiler chickens from the P1 experimental group of chickens (H&E; ×100).

**Figure 3:**
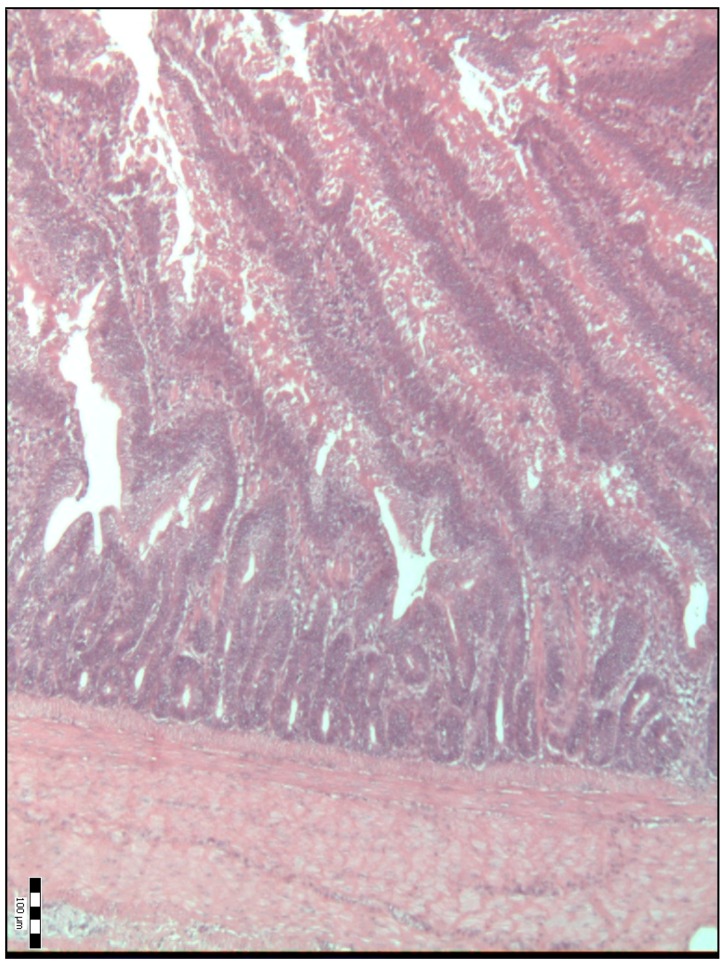
Histological representation of the duodenal villi of broiler chickens from the P2 experimental group of chickens (H&E; ×100).

**Figure 4:**
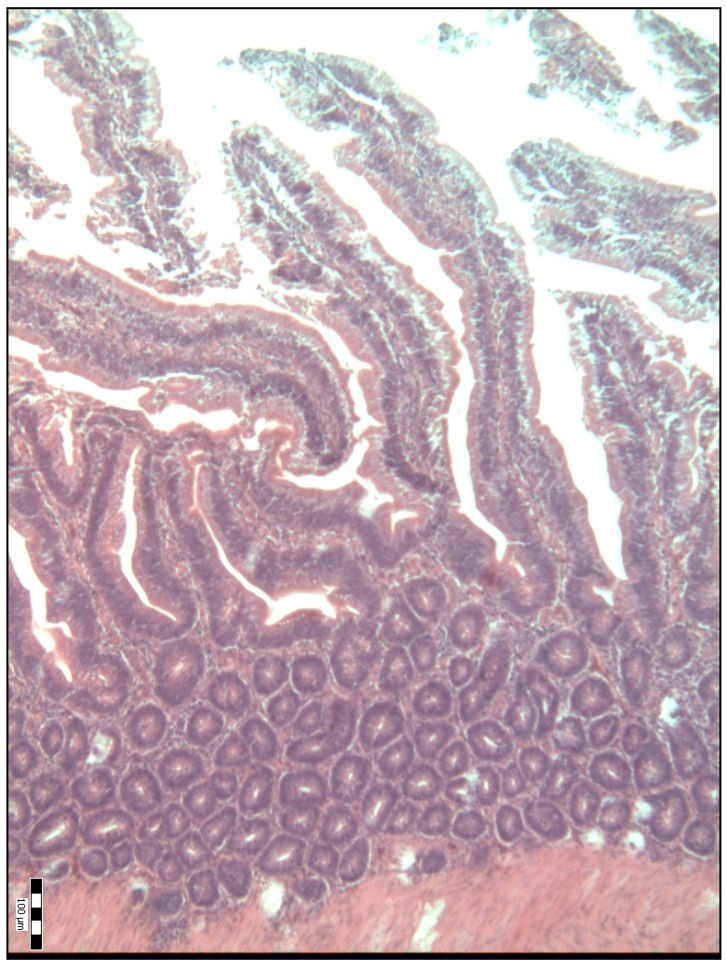
Histological representation of the duodenal villi of broiler chickens from the P3 experimental group of chickens (H&E; ×100).

**Figure 5:**
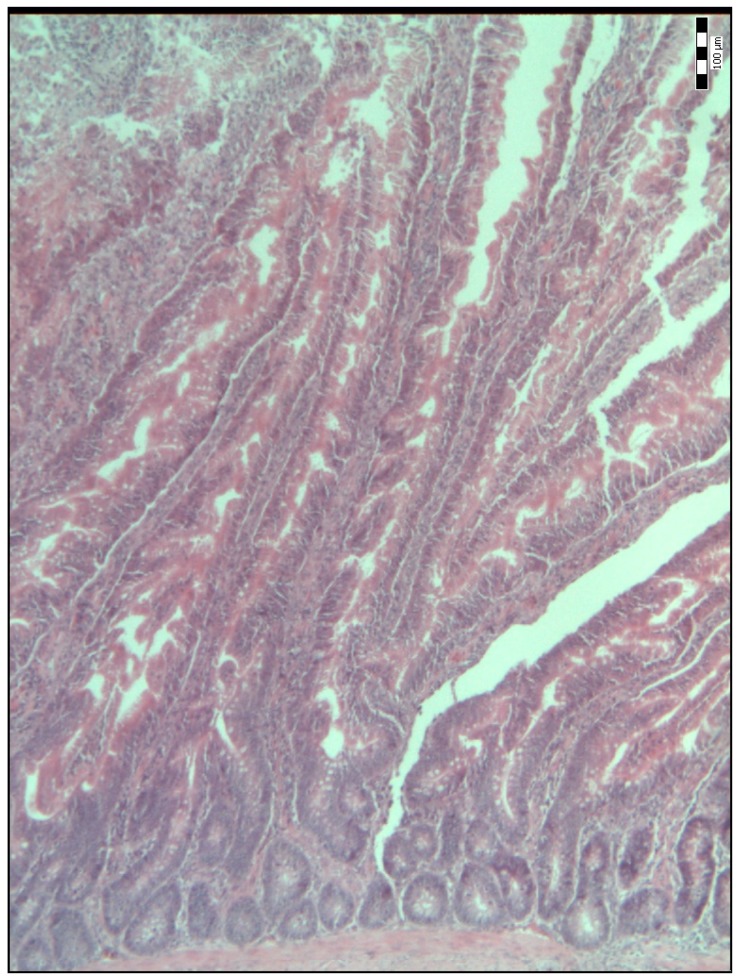
Histological representation of the duodenal villi of broiler chickens from the P4 experimental group of chickens (H&E; ×100).

The histological representations of the duodenal villi of broiler chickens from all the groups are shown in Figure 1, Figure 2, Figure 3, Figure 4 and Figure 5.

The study also revealed that there was a statistically significant difference in the villus height-to-crypt depth ratio on the 42nd day of the feeding trial between the control and experimental group of chickens (*p* < 0.001), as shown in Table 4.

**Figure 6:**
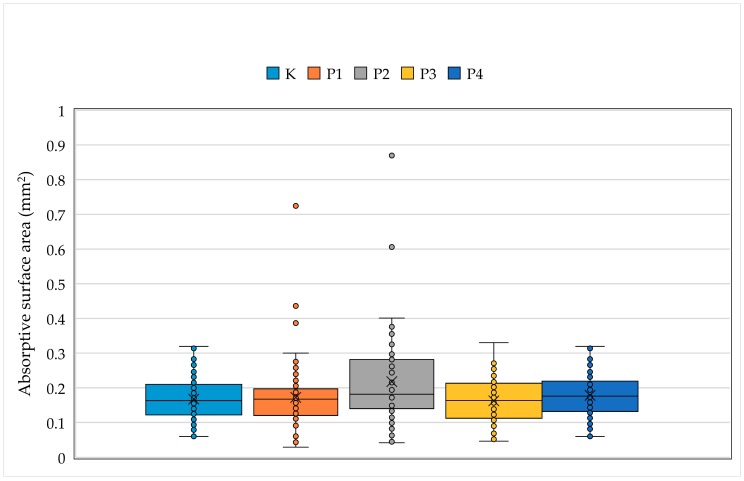
The average absorptive surface area of duodenal villi according to the group of broiler chickens on the 42nd day of the feeding trial (Kruskal–Wallis test, *p* < 0.001).

The study further showed that there was a statistically significant difference between the average values of the absorptive surface areas of the duodenal villi of broiler chickens on the 42nd day of the feeding trial between the control and experimental group of chickens (*p* < 0.001) (see Figure 6).

## 4. Discussion

Morphometric results of the duodenal villi in chickens on day 42nd of feeding trial revealed that it was significantly higher, while its base was significantly wider in the experimental groups compared to the controls. These results are consistent with the results of the study by Wang et al. [16], who demonstrated that chickens fed a diet supplemented with a mixture of bee pollen had significantly higher and wider intestinal villi of the duodenum, jejunum and ileum in comparison to the chickens fed a control diet. The same authors further determined that the observed differences were greater during the early stages of development of the gastrointestinal system [16]. The results of the present study are also consistent with the results of a study by Tekeli et al. [25], who showed that the addition of ginger and propolis extract both separately and in combination in the diet resulted in a significant increase in the length of the intestinal villi of the jejunum in chickens from the experimental groups when compared to chickens of the control group. On the other hand, Eyng et al. [17] showed that the intestinal villi of the duodenum of chickens fed a diet supplemented with various amounts of propolis were shorter or lower when compared to the intestinal villi of the chickens in the control group.

Considering the morphometric results of the duodenal villi crypt depths in chickens on the 42nd day of fattening, this study showed that there were significant differences in the depths of duodenal villi crypts between the chickens from the experimental groups compared to the chickens from the control group. This result is consistent with that of Eyng et al. [17], who showed that the crypt of the intestinal villi of the duodenum of chickens fed a diet supplemented with various amounts of propolis were deeper compared to crypt of the intestinal villi of the duodenum of chickens from the control group.

All the previously mentioned results of this study can be attributed to the beneficial effect of the biologically active components of propolis and/or bee pollen. These components participate in controlling the proliferation of pathogenic bacteria and the consequent avoidance of possible damage to the intestinal mucosa, which also leads to the reduction of morphometric measures of the intestinal villi [17,26].

Within the explanation of the identified influence of propolis and/or bee pollen on the histological features of chickens’ intestines, it is important to keep in mind that diet composition is in fact the main factor that can modify the histological appearance or morphology of the intestine and, consequently, its absorptive capacity, which ultimately defines the growth performance of fattening chickens [27]. It is further known that the intestinal villi are quickly and continuously adjusted as a response to conditions in the lumen of the intestine (that are strongly influenced by diet composition) reflecting the dynamic environment inside the intestines of animals. Accordingly, longer intestinal villi are associated with an increase in the absorptive surface of the intestines and also with an increase of the absorption capacity of the intestine [28]. This finding was also demonstrated in the present study, since the absorptive surface area of duodenal villi in all experimental groups were increased in comparison to that of the control broilers.

Previous studies have already confirmed that longer intestinal villi indicate an improved ability to absorb nutrients in the intestine [29,30]. In addition, it has been proven that longer villi are associated with active cell mitosis, which provides a greater absorptive potential of villi for various nutrients [31,32]. Deeper intestinal villi crypts indicate a rapid metabolism of tissue in order to allow the renewal of the intestinal villi, if there is a need for its regeneration [27]. Lowering the height of the villi or reducing crypt depths of intestinal villi may lead to a reduction in the absorption of nutrients [33].

This study further showed that there was a statistically significant difference in the villus height to crypt depth ratio on the 42nd day of the feeding trial between the control and experimental groups of chickens. This result is highly important, bearing in mind that a higher ratio of villous height and crypt depth refers to a greater capacity of nutrient digestibility and absorption in chickens [34]. Namely, it has been proven that shorter intestinal villi relative to crypt depth are related to a smaller number of absorptive cells and a larger number of secretory cells. Secretory cells are responsible for the secretion of mucins that form a mucinous lining of the intestinal epithelium, thus increasing the number of secretory cells and leading to an increased secretion of mucin. Changes in the quantity or composition of mucin of the intestinal mucosal surface can reduce the absorption of nutrients and/or increase the amount of energy required to maintain function of the intestines [27,35].

In present study, all the experimental groups of chickens had deeper crypts of the intestinal villi of the duodenum in relation to chickens from the control group, which is a clear indicator of higher proliferative activity in the mucosa of these intestinal villi. Higher proliferative activity in the mucosa of the intestinal villi indicates better digestibility and absorption of consumed feed mixtures in the experimental groups of chickens that were fed a mixture with the addition of propolis and/or bee pollen. The latter has also been shown in studies of other substances of pronounced antimicrobial and antioxidant properties, such as, for example, garlic and some herbal extracts [36,37,38].

The clarification of antimicrobial and antioxidant effects of all the previously mentioned substances, including propolis and bee pollen, re-emphasizes the role of their phenolic components such as various flavonoids, phenolic acids and their derivatives that they have the ability to protect the intestinal villi and increase the absorption of nutrients [38]. It is believed that these biologically active components exert their antioxidant activity both at the cellular and at the tissue level [39]. Apart from their antioxidants, their antimicrobial activity should also be significant as these bioactive agents can modulate the gut ecosystem. Due to the synergism of antioxidant and antimicrobial activity of biologically active phenolic compounds from propolis and bee pollen, a further positive effect on the utilization of nutrients has been achieved [40,41].

The present study revealed some original solutions regarding the applied dosage of investigated natural supplements and their specific combinations in broilers feeding, but was not without limitations. Due to the commonly accepted ’3Rs’, the authors had the justifiable wish to minimize the number of animals used in this study that had already been used in similar studies [3]. However, considering the tested natural feeding additives and main objective of this study, the authors believe that the described design of the study did not affect the results.

In conclusion, the present study showed that the addition of propolis and/or bee pollen to feed mixtures has a significant protective effect on the gut tissue of chickens, which is reflected through better morphometric measures of the duodenal villi and duodenal villi crypts of chickens from all the experimental groups in relation to chickens from the control group. Following the results of this study, the addition of 0.5 g of propolis per kg of feed mixture showed the strongest positive effect on chicken guts. The promising and encouraging results of this study emphasize the importance of the further evaluation of the administration level of investigated supplements in order to maximize their positive effects on the gut tissue of chickens and, consequently, the overall health of broiler chickens.

## Figures and Tables

**Table 1:** The composition and calculated analysis of feed mixtures used in the feeding of the broilers.

Ingredients, %	Starter	Finisher
	Day 1–21	Day 22–42
Corn grain	45.00	46.10
Flour middling	2.80	3.00
Dehydrated alfalfa	2.80	4.00
Soybean meal	20.20	10.00
Sunflower meal	4.00	4.00
Yeast	4.00	3.00
Full fat soybean	12.40	20.00
Vegetable oil	3.70	5.00
Monocalcium phosphate	1.20	1.20
Limestone	1.60	1.40
Salt	0.30	0.30
Premix *	1.00	1.00
Pigozen 801	1.00	1.00
Total	100.00	100.00
Calculated Analysis		
Crude protein, %	21.02	19.15
Crude fat, %	8.36	10.96
Crude fiber, %	4.96	5.05
Lysine, %	1.11	0.96
Methionine, %	0.66	0.61
Tryptophan, %	0.26	0.23
Calcium, %	1.04	0.98
Phosphorous, %	0.70	0.67
ME, MJ/kg	12.30	13.10

* Each 1 kg of premix contained: vitamin A 1200,000 IU; vitamin D3 200,000 IU; vitamin E 3000 mg; vitamin K3 250 mg; vitamin B1 150 mg; vitamin B2 600 mg; vitamin B6 200 mg; vitamin B12 1 mg; folic acid 50 mg; niacin 4400 mg; Ca pantothenate 1500 mg; biotin 10mg; choline chloride 50,000 mg; iron 5000 mg; copper 700 mg; manganese 8000 mg; zinc 5000 mg; iodine 75 mg; cobalt 20 mg; magnesium 750 mg; selenium 15 mg; antioxidant butylated hydroxytoluene (BHT) 10,000 mg; methionine 100,000 mg; herbal carrier 1000 g.

**Table 2:** The amount of total flavonoids (mg/g) in propolis and bee pollen, expressed as equivalents of quercetin.

**The Amount of Total Flavonoids (mg/g), Expressed as Equivalents of Quercetin**	**Propolis**	**Bee Pollen**
	248.24	31.80

**Table 3:** The values of evaluated parameters of duodenal villi of broiler chickens on the 42nd day of the feeding trial.

Parameter	Group of Chickens Median (Q1–Q3)					* *p*
	K	P1	P2	P3	P4	
Duodenal villi height (μm)	718.50 ^a^ (584.50–841.50)	834.00 ^b^ (695.00–990.00)	992.00 ^c^ (814.50–1111.50)	886.00 ^bcd^ (697.00–1134.50)	798.50 ^bde^ (658.75–1088.00)	<0.001
Base width of the duodenal villi (μm)	48.00 ^a^ (38.00–68.00)	59.00 ^b^ (52.00–69.50)	67.00 ^bc^ (51.00–77.25)	54.00 ^bd^ (45.50–67.00)	65.00 ^ce^ (58.75–75.25)	<0.001
Duodenal villi crypt depth (μm)	78.00 ^a^ (66.00–93.00)	85.00 ^a^ (75.50–93.50)	71.50 ^b^ (63.75–80.25)	64.00 ^c^ (54.00–74.50)	78.00 ^ad^ (70.00–85.25)	<0.001

* Kruskal–Wallis test. abcde: Medians within a row with different superscripts are different; K = control group; P1 = feed mixture + 0.25 g of propolis/kg of feed mixture + 20 g of bee pollen/kg of feed mixture; P2 = feed mixture + 0.5 g of propolis/kg of feed mixture; P3 = feed mixture + 1.0 g of propolis/kg of feed mixture; P4 = feed mixture + 20 g of bee pollen/kg of feed mixture.

**Table 4:** The villus height-to-crypt depth ratio of broiler chickens on the 42nd day of the feeding trial.

Parameter	Group of Chickens Median (Q1–Q3)					* *p*
	K	P1	P2	P3	P4	
**The Villus Height-to-Crypt Depth Ratio**	8.86 ^a^ (7.16–10.60)	9.81 ^b^ (8.51–12.09)	14.24 ^c^ (11.64–16.36)	13.61 ^cd^ (10.54–16.70)	10.89 ^be^ (8.70–12.73)	<0.001

* Kruskal–Wallis test. (Q1–Q3) = interquartile range; K = control group; P1 = feed mixture + 0.25 g of propolis/kg of feed mixture + 20 g of bee pollen/kg of feed mixture; P2 = feed mixture + 0.5 g of propolis/kg of feed mixture; P3 = feed mixture + 1.0 g of propolis/kg of feed mixture; P4 = feed mixture + 20 g of bee pollen/kg of feed mixture.
